# A New Antifibrotic Target of Ac-SDKP: Inhibition of Myofibroblast Differentiation in Rat Lung with Silicosis

**DOI:** 10.1371/journal.pone.0040301

**Published:** 2012-07-03

**Authors:** Hong Xu, Fang Yang, Ying Sun, Yuan Yuan, Hua Cheng, Zhongqiu Wei, Shuyu Li, Tan Cheng, Darrell Brann, Ruimin Wang

**Affiliations:** 1 Department of Pathology, Hebei Medical University, Shi Jiazhuang, China; 2 Medical Research Center, Hebei United University, Tangshan, China; 3 Institute of Molecular Medicine and Genetics, Georgia Health Sciences University, Augusta, Georgia, United States of America; Helmholtz Zentrum München/Ludwig-Maximilians-University Munich, Germany

## Abstract

**Background:**

Myofibroblast differentiation, characterized by α-smooth muscle actin (α-SMA) expression, is a key process in organ fibrosis, and is induced by TGF-β. Here we examined whether an anti-fibrotic agent, N-acetyl-seryl-aspartyl-lysylproline (Ac-SDKP), can regulate induction of TGF-β signaling and myofibroblast differentiation as a potential key component of its anti-fibrotic mechanism in vivo and in vitro.

**Methodology/Principal Findings:**

Rat pulmonary fibroblasts were cultured in vitro and divided to 4 groups 1) control; 2) TGF-β1; 3) TGF-β1+ LY364947; 4) TGF-β1+Ac-SDKP. For in vivo studies, six groups of animals were utilized 1) control 4w; 2) silicotic 4w; 3) control 8w; 4) silicotic 8w; 5) Ac-SDKP post-treatment; 6)Ac-SDKP pre-treatment. SiO_2_ powders were douched in the trachea of rat to make the silicotic model. Myofibroblast differentiation was measured by examining expression of α-SMA, as well as expression of serum response factor (SRF), a key regulator of myofibroblast differentiation. The expressions of collagen, TGF-β1 and RAS signaling were also assessed. The results revealed that TGF-β1 strongly induced myofibroblast differentiation and collagen synthesis in vitro, and that pre-treatment with Ac-SDKP markedly attenuated myofibroblast activation, as well as induction of TGF-β1 and its receptor. Similar results were observed in vivo in the pathologically relevant rat model of silicosis. Ac-SDKP treatment in vivo strongly attenuated 1) silicosis-induced increased expressions of TGF-β1 and RAS signaling, 2) myofibroblast differentiation as indicated by a robust decrease of SRF and α-SMA-positive myofibroblast localization in siliconic nodules in the lung, 3) collagen deposition.

**Conclusion/Significance:**

The results of the present study suggest a novel mechanism of action for Ac-SDKP’s beneficial effect in silicosis, which involves attenuation of TGF-β1 and its receptors, SRF and Ang II type 1 receptor (AT_1_) expression, collagen deposition and myofibroblast differentiation. The results further suggest that therapies targeting myofibroblast differentiation may have therapeutic efficacy in treatment of silicosis of the lung.

## Introduction

Silicosis, a common occupational respiratory disease, is a pathological condition of the lungs due to inhalation of particulate matter containing crystalline silica. Although advances in occupational safety and health make this disorder highly preventable, silicosis remains the most prevalent occupational disease worldwide. In China, the number of cases has increased rapidly due to the expansive growth of industry and the absence of available methods to prevent dust [1], [2]. By the end of 2010, China recorded 676,541 total cases of pneumonoconiosis (approximately 10,000 new cases per year), with half of the cases being silicosis [3]. Furthermore, there is no therapy for silicotic disease in general, largely because the underlying basis of fibrosis is unclear.

Although many different cell types and cytokines have been implicated to have a role in fibrotic diseases, there is increasing evidence that myofibroblasts, which express α-smooth muscle actin (α-SMA), represent a principal effector cell in lung fibrosis [4]. Furthermore, there is significant evidence from our group and other supporting transforming growth factor-β (TGF-β) as a key modulator of lung fibrosis [5], [6]. Responses elicited by TGF-β are dependent on and specific for the target cell lineage, and are classically mediated by receptor-mediated intracellular signaling pathways that involve Smad proteins. Mechanistically, TGF-β-Smad 2/3 signaling has been shown to induce a genetic program that leads to disproportionate increases in collagen and fibronectin expression, and promotes extracellular matrix (ECM) by decreasing secretion of proteases and increasing secretion of protease inhibitors [7]. TGF-β signaling alters the fibroblast phenotype by promoting its differentiation into morphologically distinct pathological myofibroblasts characterized by the expression of α-SMA [8], [9], [10] which, in turn, promotes collagen synthesis and enhanced ECM deposition. The role of myofibroblastic differentiation and the acquisition by fibroblasts of smooth muscle-like phenotypic features in wound healing and fibrosis has been extensively reviewed recently [11], [12], [13]. In vascular smooth muscle cells, SMA-gene transcription is controlled primarily through the binding of serum response factor (SRF) to its target cis-elements, the CArG boxes (CC(AT)_6_GG) within the promoter regions of most SMA genes, including α-SMA [14]. Furthermore, it has been reported that TGF-β1 induces expression and activation of serum response factor (SRF), which is required for myofibroblast differentiation [15].

With respect to anti-fibrotic factors, N-acetyl-seryl-aspartyl-lysylproline (Ac-SDKP) is a natural anti-inflammatory and anti-fibrotic peptide [16]. Previously, we and others have found that Ac-SDKP inhibits organ fibrosis, such as heart, renal and lung fibrosis [6], [17], [18]. It is hydrolyzed exclusively by angiotensin-converting enzyme (ACE), and its plasma concentration is increased substantially by ACE inhibitors. Furthermore, local renin-angiotensin systems (RAS) have been described as an important regulator of the fibrotic response to tissue injury as well as TGF-β. Activation of RAS and production of angiotensin II (Ang II) is associated with tissue fibrosis [19], [20]. Recent studies indicate that Ang II and TGF-β1 act as part of an integrated signaling network that promotes cardiac remodeling and possibly fibrosis [21], [22], [23], [24].

Work by our group has demonstrated that Ac-SDKP can inhibit pulmonary fibrosis in rats with SiO_2_-induced silicosis by inhibiting chronic inflammation, TGF-β1/Smad signal, and TGF-β1 induced pulmonary fibroblast proliferation and collagen synthesis [6], [25], [26]. However, it is unknown whether Ac-SDKP can modulate myofibroblast differentiation by regulating SRF expression in vivo and in vitro, and whether it can modulate TGF-β and RAS signaling in the rat silicotic model. Thus, the purpose of the current study was to address these key unanswered questions.

## Materials and Methods

### Preparation and Culture of Fibroblasts

Rat lung fibroblasts were isolated from 1–3 days old Wistar rats according to a previously described procedure [6]. Cells were maintained in a complete culture medium [Dulbecco’s modified Eagle’s medium (DMEM, GIBCO) supplemented with 10% fetal bovine plasma (FBS, PAA), 100 U/ml penicillin/streptomycin] in a 37°C incubator with 5% CO_2_. At confluence, cells were split with 0.25% trypsin at the ratio 1∶3 every passage. Cells from 3 to 6 passages were used in this study. When cells reached sub-confluence, they were cultured in DMEM containing 0.5% FBS for 24 hours and most cells were in quiescent state. Then, they were divided into 4 groups and cultured for 48 hours: 1) control (0.5%-FBS DMEM); 2) TGF-β1 (5 µg/ml, Peprotech) + vehicle; 3) TGF-β1+ LY364947 (59 nM, Cayman); 4) TGF-β1+ Ac-SDKP (10^−8^ M). The cells were treated with LY364947 and Ac-SDKP for 1 hour before TGF-β1 induction.

### In vivo Experimental Protocol and Disease Model

Male Wistar rats, weighting 180±10 g, were purchased from Vital River Laboratory Animal Technology Co. Ltd. (Beijing, China). The animal experiment was reviewed and approved by the Institutional Animal Care and Use Committee at the Hebei United University. Animals received food and water according to guidelines set by the National Institute of Health (NIH). The rats were anesthetized with Isoflurane and received either silica solution (50 mg/rat, 1 ml) or 0.9% saline (vehicle) by trachea instillation. Prior to instillation, the 5 µm silica particles (Sigma, St. Louis, MO, USA) were baked at 180°C for 6 hours. Ac-SDKP [800 µg/(kg d), Bachem AG company, USA] or control (0.9% saline) was given via a mini-osmotic pump (Model 2 ml4, DURECT Co. Ltd, USA) implanted into the abdominal cavity. Rats were divided into 6 groups: 1) control 4w (instilled with 0.9% saline and then treated with 0.9% saline for 4w); 2) silicotic model 4w (instilled of SiO_2_, and then treated with 0.9% saline for 4w); 3) control 8w (treated with 0.9% saline 48 h before 0.9% saline instillation and then continued treatment for 8w.); 4) silicotic model 8w (treated with 0.9% saline 48 h before SiO_2_ instillation and then continued treatment for 8w); 5) Ac-SDKP post-treatment (instilled with SiO_2_ and treated with 0.9% saline for 4w and Ac-SDKP for another 4w); 6) Ac-SDKP pre-treatment (treated with Ac-SDKP 48 h before instillation of SiO_2_, and then continued for 8w). Each experimental group included ten animals. The histopathology (H.E. stain) and hydroxyproline assay were determined as described previously [6].

### Immunocytochemistry and Immunohistochemistry for α-SMA and SRF

Cells growing on glass slide [27] and paraffin-embedded sections were permeabilized with 0.2% Triton and blocked with 5% bovine plasma albumin (BSA) in 0.1 M phosphate-buffer saline (PBS) for 30 minutes to reduce nonspecific binding, then were incubated with primary antibodies against α-SMA (1184-s, EPIT MICs Biotechnology, USA) and SRF (sc-335, Santa Cruz Biotechnology, USA) followed by the biotinylated secondary antibody and finally the ABC reagent (Wuhan Boshide Biological Engineering Co. Ltd, China). Immunoreactivity was visualized with DAB. A brown color staining was considered a positive result.

### ELISAs

The plasma concentrations of TGF-β1 and Ang II were determined by Enzyme-Linked ImmunoSorbent Assay (ELISA) following the suggested manufacturer’s protocol (NeoBioscience Technology and CusaBio Biotech, CSB-E04494r). The drug level of Ac-SDKP in lung tissues was assayed by ELISA kits (CSB-E009265r, CusaBio Biotech). Briefly, 100 mg lung tissue was rinsed with 0.01 M PBS, homogenized in 1 ml of PBS and stored overnight at −20°C After two freeze-thaw cycles were performed to break the cell membranes, the homogenates were centrifuged for 5 mins at 5000 g. The assay minimum detectable concentration of TGF-β1, Ang II and Ac-SDKP was 31.25 pg/ml, 1.17 pg/ml and 0.078 ng/ml, respectively.

### Western Blot Analysis

The cells and lung tissue were lysed by RIPA (ZO2338A, Aidlab; China), and the total protein content was quantified by a protein assay (PC0020, Solarbio; China). The proteins (70 µg/lane) were separated in 10% gel by SDS-PAGE and electrotransferred to a nitrocellulose membrane (Amervehicle Biosciences). Membranes were blocked with 5% non-fat milk and incubated overnight at 4°C with the primary antibody [anti-collagen type I (C2456) and III (C7805), Sigma; anti-TGF-β1 and receptor, (Wuhan Boshide Biological Engineering Co. Ltd, China); anti-ACE (2504-1) and anti- Ang II type 1 receptor (AT_1_; 5172-1), EPIT MICS, USA; anti-Ang II(bs-0578R), Beijing Boisynthesis Biotechnology co. LTD, China; anti-α-SMA and anti-SRF; anti-β-actin (sc-47778), anti-GAPDH (sc-25778), Santa Cruz Biotechnology] followed by alkaline phosphatase-conjugated secondary antibodies (E030220, E020210, EarchOx, USA). Target bands were visualized by addition of BCIP/NET (E116, Amresco, USA). Results were normalized with GAPDH or β-actin and expressed as the fold of specific bands to the control 4w group.

### Quantitative Real Time Polymerase Chain Reaction (PCR)

The following oligonucleotide primers specific for rat genes were used in this study: collagen type I, sense 5′-CAGATTGGGATGGAGGGAGTTTA-3′ and antisense 5′-CTACAGCACGCTTGTGGA TGGCT-3′; collagen type III, sense 5′-ATAGCTGAACTGAAAGCCACCAT-3′ and antisense 5′-CCTGAACTCAAGAGCGGAATA-3′; α-SMA, sense 5′-TCCAGAGTCCAGCACAATACCAG-3′ and antisense 5′-AATGACCCAGATTATGTTTGAGACC-3′; SRF, sense 5′-CTTAACATGGCATC TTCGACACT-3′ and antisense 5′-CTTAACCTCTAATCCCCATTGCT-3′; GAPDH, sense 5′-GTCACCTTCACCGTTCCAGTTTT-3′ and antisense 5′-CTTAGTTGCGTTACACCCTTTCTT-3′. Total RNA was extracted from fibroblasts using Trizol reagent (Invitrogen), and cDNA was generated from 1 µg RNA using a random hexamer and the M-MLV first strend kit (c28025–032, Invitrogen). Real time PCR was performed as described in the PCR core kit of SBYR green (c11733–038, Invitrogen). The data were analyzed using the ΔΔCt method and presented as arbitrary units.

### Statistical Analysis

Values were expressed as mean±SEM. Comparisons between multiple independent groups were conducted using one-way ANOVA followed by post hoc analysis with the Bonferroni test. Group differences resulting in p-values of less than 0.05 were considered to be statistically significant.

## Results

### Effect of Ac-SDKP on TGF-β Receptor Type I and II in Cultured Rat Pulmonary Fibroblasts Induced by TGF-β1

**Figure 1:**
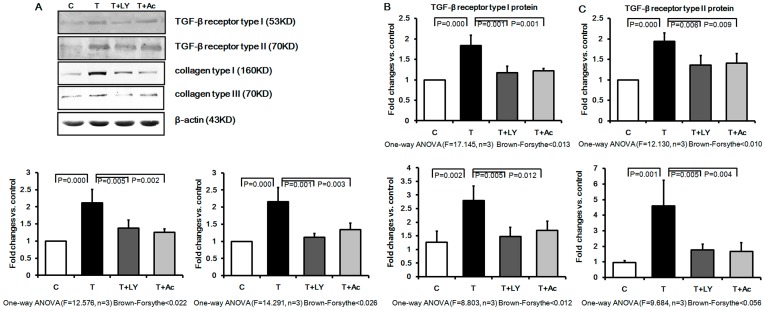
Effect of Ac-SDKP on expression of TGF-β receptor type I and II, collagen type I and III in cultured rat fibroblasts. TGF-β treatment of cultured rat fibroblasts enhanced protein and mRNA levels of TGF-β receptor and increased collagen synthesis, while pretreatment with Ac-SDKP and LY364947 markedly attenuated the effects of TGF-β1. A) Western blot analysis with antibodies against TGF-β receptor type I and II, collagen type I and III; C: control group; T: TGF-β group; T+ LY: TGF-β+ LY364947 group; T+ Ac: TGF-β+ Ac-SDKP group. B) The TGF-β receptor type I protein expression measured by Western blot; C) The TGF-β receptor type II protein expression measured by Western blot; (D) The collagen type I protein expression measured by Western blot; (E) The collagen type III protein expression measured by Western blot; F) The collagen type I mRNA expression measured by Real-time PCR; G) The collagen type III mRNA expression measured by Real-time PCR.

As shown in Figure 1 A–C, Western blot analysis revealed that TGF-β1 treatment of cultured rat pulmonary fibroblasts resulted in up-regulation of the TGF-β receptor type I and II by 1.7 and 1.8 fold, respectively, as compared to non-induced cells. Furthermore, treatment with LY364947, a TGF-β receptor inhibitor, attenuated the up-regulation effect induced by TGF-β1. Finally, pre-treatment with the anti-inflammatory agent, Ac-SDKP induced a similar decrease of the TGF-β receptor type I and II expression to 76.83% and 84.92%, respectively.

### Effect of Ac-SDKP on Protein and mRNA Levels of Collagen Synthesis in Cultured Rat Pulmonary Fibroblasts Induced by TGF-β

We next examined expression of collagen type I and III after TGF-β induction to elucidate the effect of Ac-SDKP on extracellular matrix (ECM) synthesis. Following induction by TGF-β1, collagen type I and III protein levels were significantly increased in the cultured pulmonary fibroblasts as compared to the control group (Fig. 1 A, D and E). In contrast, pretreatment with Ac-SDKP resulted in significant attenuation of collagen type I and III expression. In addition, LY364947 significantly attenuated TGF-β1-induced expression of collagen type I and III. Furthermore, TGF-β1 induced a significant up-regulation of collagen type I and III mRNA levels as compared to control cells, and this effect was also significantly inhibited by pretreatment with either LY364947 or Ac-SDKP (Figure 1 F, G).

### Effects of Ac-SDKP on Myofibroblast Differentiation in Cultured Rat Pulmonary Fibroblasts Induced by TGF-β1

**Figure 2:**
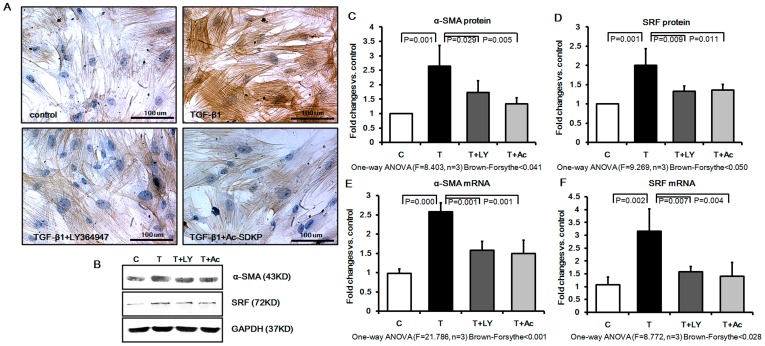
Effect of Ac-SDKP on expression of α-SMA and SRF in cultured rat fibroblasts. TGF-β up-regulated the expression of α-SMA in fibroblasts to promote the differentiation from fibroblast to myofibroblast. In contrast, Ac-SDKP and LY364947 pretreatment prevented the α-SMA and SRF expression. A) α-SMA protein expression and distribution as analyzed by immunohistochemistry; B) Western blot analysis with antibodies against α-SMA and SRF; C) α-SMA protein expression measured by Western blot; D) SRF protein expression measured by Western blot; E) α-SMA mRNA expression measured by Real-time PCR; F) SRF mRNA expression measured by Real-time PCR.

The effects of TGF-β1 and Ac-SDKP on myofibroblast differentiation were examined by accessing their effects upon the myofibroblast differentiation marker, α-SMA, and upon serum response factor (SRF), a key factor that regulates myofibroblast differentiation. As shown in Figure 2A, TGF-β1 induced a marked change in fibroblast morphology after 48 hours of treatment, with a larger cell body and parallel or overlapped spindle-shaped actin fibers, and dramatically increased α-SMA staining as visualized by microscopy. Pre-treatment with LY364947 and Ac-SDKP was able to significantly attenuate these changes. Western blot analysis further confirmed the immunocytochemistry results by showing that Ac-SDKP and LY364947 pretreatment induced a significant reduction of α-SMA to 51.54% and 68.08% of that in the TGF-β1 treated group, respectively (Figure 2, B and C). Likewise, Western blot analysis confirmed a strong up-regulation of SRF expression in TGF-β1 treated fibroblasts as compared to control cells, and that this effect was significantly inhibited by pretreatment with either LY364947 or Ac-SDKP (Figure 2, B and D). Furthermore, as shown in Figure 2 D and E, SRF and α-SMA mRNA levels in the cultured fibroblasts were also significantly increased by TGF-β1, and this up-regulation was significantly inhibited by pretreatment with either LY364947 or Ac-SDKP (Figure 2, E and F).

### Effect of Ac-SDKP on Myofibroblast Differentiation in Rat Silicotic Model

**Figure 3:**
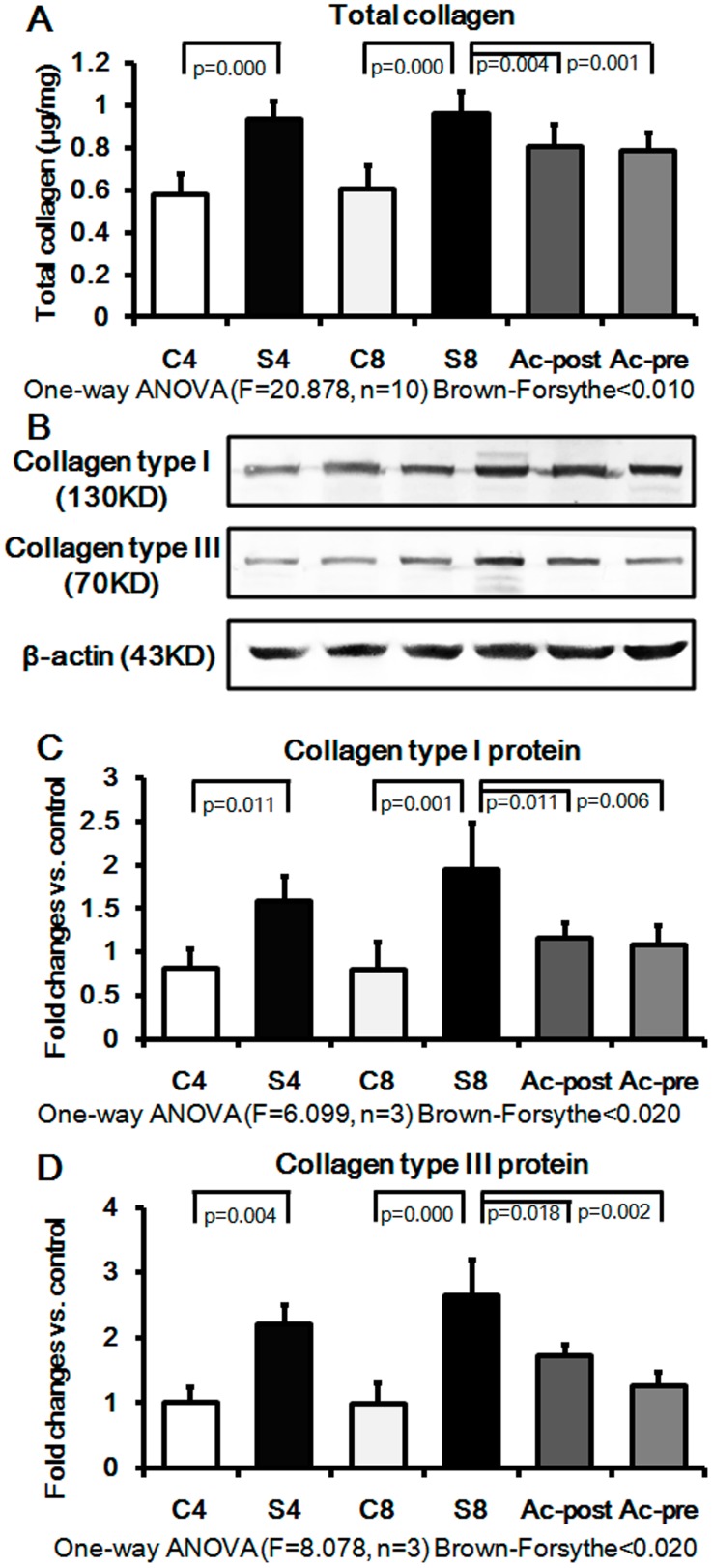
Effect of Ac-SDKP on lung collagen expression in the rat silicotic model. Compared with control group, the lung collagen content was significantly increased in the rat silicotic model, while Ac-SDKP treatment induced a significant decrease of collagen expression. A) Total collagen content measured by hydroxyproline assay; C4: control 4w group; S4: silicotic model 4w group; C8: control 8w group; S8: silicotic model 8w group; Ac-post: Ac-SDKP post-treatment group; Ac-pre: Ac-SDKP pre-treatment group; B) The tissue extracts were analyzed by Western blot analysis with antibodies against collagen type I and III; C) The collagen type I protein expression measured by Western blot; D) The collagen type III protein expression measured by Western blot.

We next sought to determine whether Ac-SDKP can regulate myofibroblast differentiation in a pathologically relevant *in vivo* model of silicosis. Specifically, we examined whether Ac-SDKP could suppress expression of TGF-β1 and its receptors, SRF, α-SMA and ECM in an in vivo silicotic model described previously by our group and others [6], [25]. The expression of collagen levels in the silicotic model (4w and 8w) was higher than that in control group (4w and 8w), as assessed by hydroxyproline assay and Western blot analysis (Figure 3, A–D). Interestingly, Ac-SDKP post-treatment in 8w animals markedly reduced the number of silicotic nodules, as well as total collagen, collagen type I and III (e.g. reduced to 84.08%, 59.79%, and 65.15%, respectively). Furthermore, Ac-SDKP pre-treatment was able to significantly attenuate the silicosis-induced increases in ECM deposition in the lung, and reduced total collagen, collagen type I and III were to 81.85%, 55.67%, and 47.72%, respectively.

**Figure 4:**
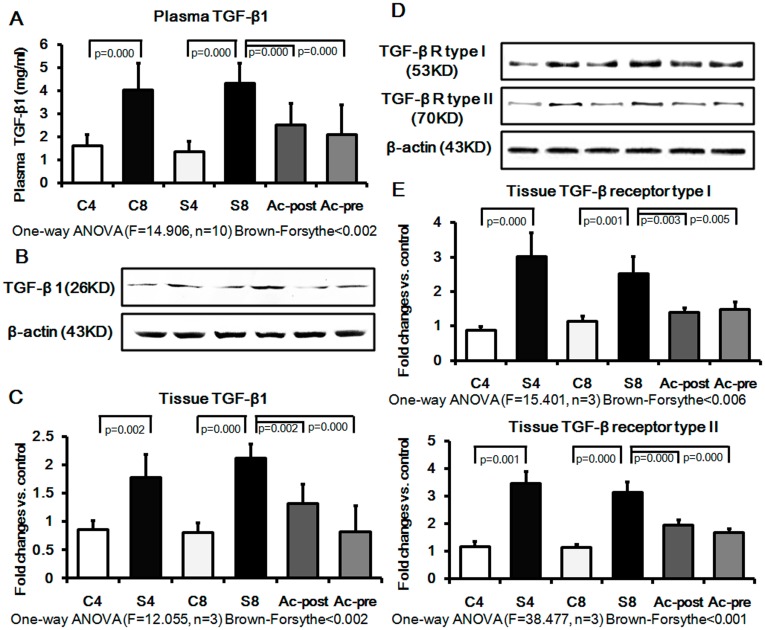
Effect of Ac-SDKP on expression of TGF-β1 and its receptor in plasma and lung of rat silicotic model. Expression of TGF-β1 and its receptor were significantly increased in the silicotic model as compared to control. After the intervention of Ac-SDKP, the expression of these proteins decreased significantly. A) The plasma level of TGF-β1 measured by ELISA; C4: control 4w group; S4: silicotic model 4w group; C8: control 8w group; S8: silicotic model 8w group; Ac-post: Ac-SDKP post-treatment group; Ac-pre: Ac-SDKP pre-treatment group; B) The tissue extracts were analyzed by Western blot analysis with antibodies against TGF-β1 and its receptor; C) TGF-β1 expression protein expression measured by Western blot. D) TGF-β receptor type I protein expression measured by Western blot; C) TGF-β receptor type II protein expression measured by Western blot.

We next examined the effects of Ac-SDKP on regulation of TGF-β1 and its receptors (type I and II) in the silicotic model. TGF-β1 levels in the plasma were significantly elevated in silicosis model (4w and 8w), and inhibited by either pre- or post-treatment with Ac-SDKP (Figure 4 A). As shown in Figure 4 B–F, the expressions of TGF-β1, TGF-β1 receptor type I and type II in silicotic model 4w increased by 2.09, 3.46 and 2.97 fold, respectively, compared with the control 4w group; and increased by 2.62, 2.07 and 2.73 fold in silicotic 8w group, as compared with the control 8w group. The up-regulation of TGF-β1 and its receptors observed in silicotic model 8w was significantly reversed by Ac-SDKP post-treatment by 62.26%, 58.09% and 62.62% of silicotic model 8w, respectively. Ac-SDKP pretreatment decreased the expression of TGF-β1, TGF-β1 RI and TGF-β1 RII by 38.68%, 61.83% and 53.85% of silicotic model 8w, respectively.

**Figure 5:**
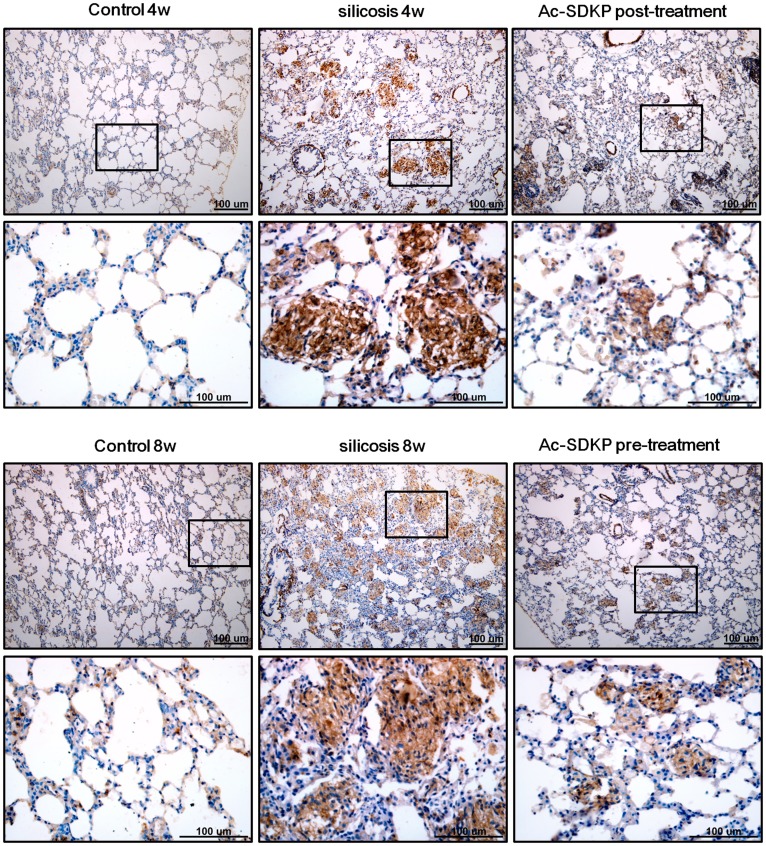
Effect of Ac-SDKP on expression α-SMA as analyzed by immunohistochemistry in the rat silicotic model. α-SMA-positive immunostaining was only observed in smooth muscle cells in vessels and trachea in control. Notably, there was more positive expression of α-SMA in silicotic nodules and the area of mesenchyma fibrosis in silicotic model (4w and 8w). Positive expression of α-SMA in Ac-SDKP post- and pre-treatment was less than that in the silicotic model.

**Figure 6:**
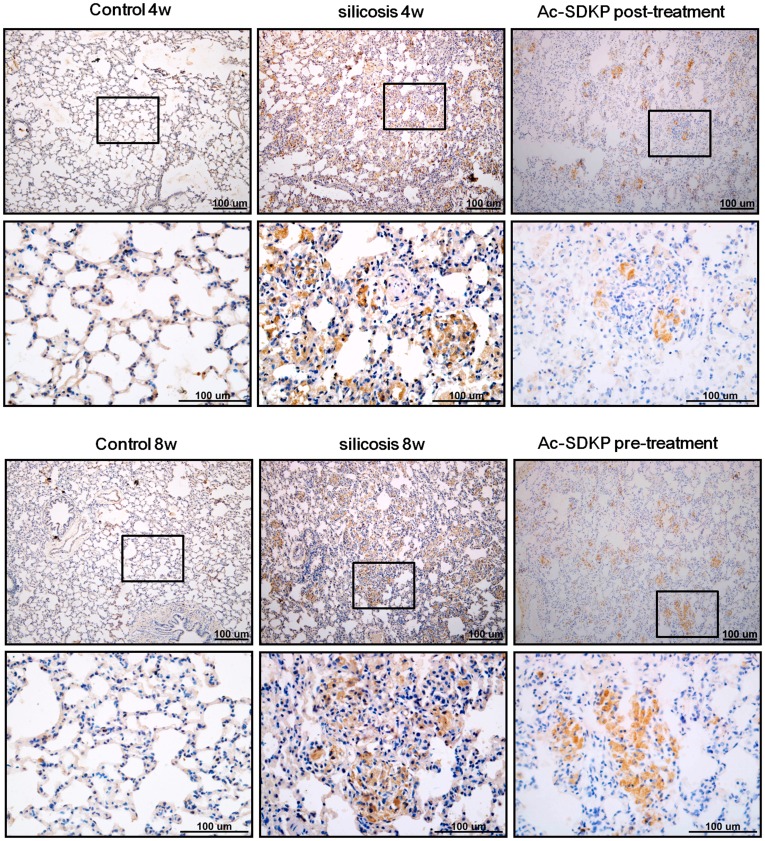
Effect of Ac-SDKP on expression of SRF as analyzed by immunohistochemistry in the rat silicotic model. SRF-positive immunostaining was only observed in smooth muscle cells in vessels and trachea in control. Notably, there was more positive expression of SRF in silicotic nodules and the area of mesenchyma fibrosis in the silicotic model (4w and 8w). Positive expression of SRF in Ac-SDKP post- and pre-treatment was less than that in the silicotic model.

**Figure 7:**
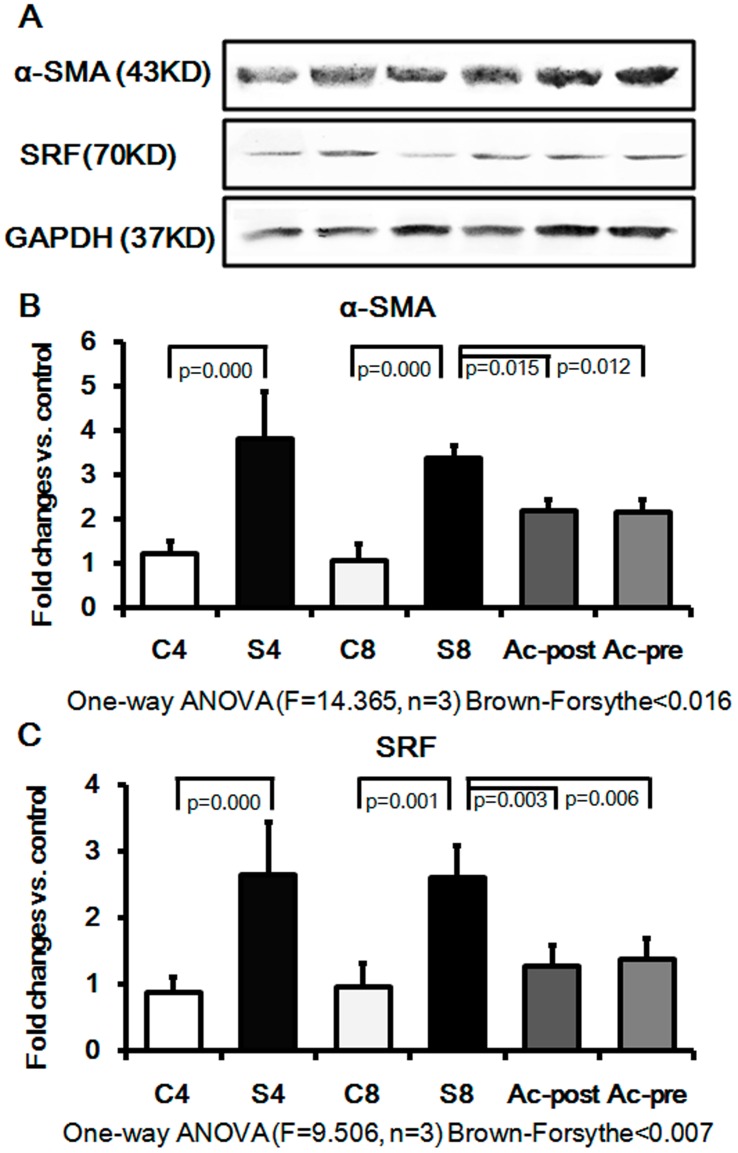
Effect of Ac-SDKP on expression of α-SMA and SRF in rat silicotic model. Expression of α-SMA and SRF was significantly increased in the silicotic model (4w and 8w) as compared to control (4w and 8w). After the intervention of Ac-SDKP, the expression of these proteins decreased significantly. A) The tissue extracts were analyzed by Western blot analysis with antibodies against α-SMA and SRF; B) The α-SMA expression protein expression measured by Western blot. C4: control 4w group; S4: silicotic model 4w group; C8: control 8w group; S8: silicotic model 8w group; Ac-post: Ac-SDKP post-treatment group; Ac-pre: Ac-SDKP pre-treatment group; C) The SRF protein expression measured by Western blot.

Induction of interstitial myofibroblasts was next assessed by immunohistochemical detection of α-SMA and SRF in the silicotic model. As shown in Figures 5,6, positive staining for α-SMA and SRF was seen in vascular vessels and trachea smooth muscle cells, but not in the interstitial space in the control group. Expression of α-SMA and SRF was also observed in silicotic nodules and interstitial fibrotic regions in the silicosis model. Of significant interest, Ac-SDKP post- and pre-treatment markedly reduced the appearance of α-SMA and SRF in the silicotic nodules and interstitial fibrotic area. The immunohistochemical results were further confirmed by Western blot analysis (Figure 7A–C), which showed that silica (4w and 8w) increases the expressions of α-SMA by 3.11 and 3.13 fold compared with control group (4w and 8w), and that Ac-SDKP post-treatment and pre-treatment significantly attenuated the expression of α-SMA in rat lung with silicosis to 66.96% and 63.69%, respectively. Similar to the expression of α-SMA, the up-regulation of SRF observed in silicotic model 8w group was attenuated to 54.02% and 53.63% in Ac-SDKP post- and pre-treatment groups, as compared with the silicotic 8w group, respectively.

### Effect of Ac-SDKP on RAS Signaling in Rat Silicotic Model

**Figure 8:**
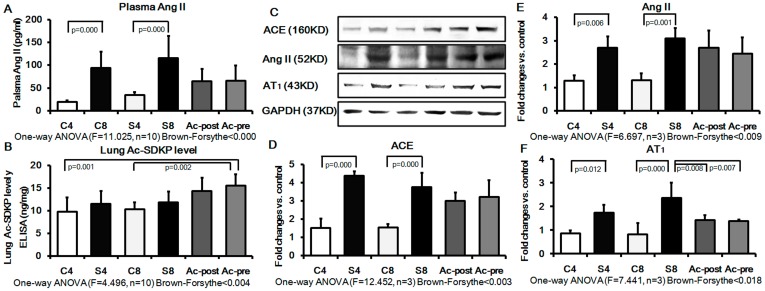
The RAS signal and Ac-SDKP in plasma and lung of rat silicotic model. Expression of ACE, Ang II and AT_1_ was significantly increased in the silicotic model (4w and 8w) as compared to control (4w and 8w). Ac-SDKP post- or pre-treatment showed a pattern to decrease the silicosis-induced expression of ACE, Ang II, and AT_1_, but only inhibition of AT_1_ expression was statistically significant. A) The plasma level of Ang II measured by ELISA; C4: control 4w group; S4: silicotic model 4w group; C8: control 8w group; S8: silicotic model 8w group; Ac-post: Ac-SDKP post-treatment group; Ac-pre: Ac-SDKP pre-treatment group; B) The level of Ac-SDKP in the lung was measured by ELISA; C) The tissue extracts were analyzed by Western blot analysis with antibodies against ACE, Ang II and AT_1_; D) The ACE protein expression measured by Western blot. E) The Ang II protein expression measured by Western blot. F) The AT_1_ protein expression measured by Western blot.

Because Ac-SDKP is hydrolyzed in the presence of ACE, we sought to determine the expression of ACE, Ang II and AT_1_ in plasma or lung tissue of rats. The results revealed that the concentration of Ang II in plasma was increased in silicotic model (4w and 8w) by 4.94 and 3.71 folds, respectively, as compared with the control group (4w and 8w). In the Ac-SDKP post-treatment and pre-treatment groups, the Ang II expression in plasma showed a pattern for decrease, which however was not statistically significant (Figure 8A). Examination of the lung tissue level of Ac-SDKP by ELISA revealed that Ac-SDKP levels were higher in Ac-SDKP post- and pre-treatment group, but the levels were not statistically significant when compared with the silicotic 8w group (Figure 8B). Ac-SDKP levels in the Ac-SDKP pre-treatment group were significantly different from the control groups (4w and 8w). Western blot analysis further confirmed the ELISA results by showing that silica increases the expression of ACE, Ang II, AT_1_ in silicotic model 4w (e.g. increased by 2.10, 2.86 and 2.20 fold, respectively, compared with the control 4w group). Expression of ACE, Ang II, AT_1_ in the silicotic model 8w were also increased by 2.34, 2.44 and 2.62 fold, respectively, compared with the control 8w group. Ac-SDKP pre- and post-treatment groups showed a significant inhibition of the silica-induced elevation of AT_1_ expression. Mean levels for ACE and AngII were generally lower in the Ac-SDKP pre- and post-treatment groups, but this effect was not statistically significant.

## Discussion

The current study advances the field by demonstrating that Ac-SDKP has an anti-fibrotic effect on silicosis in vivo and in vitro, an effect that involves inhibition of TGF-β induced myofibroblast differentiation, leading to a decrease in SRF, α-SMA and ECM deposition. Previous work had shown that pulmonary fibroblasts are key cells in the development, prevention and treatment of lung fibrosis, since interstitial fibroblast proliferation and collagen synthesis play an essential role in the occurrence and development of lung silicosis. Intriguingly, during the development of fibrosis, there is differentiation of myofibroblasts, whose shape and feature are between fibroblasts and smooth muscle cells in the fibrotic area, and which expresses α-SMA as its hallmark feature. The source of myofibroblasts is not well understood, but fibrocytes, including local fibroblasts, epithelial cells, endothelial cells, smooth muscle cells, pericytes, hepatic perisinusoidal cells, mesenchymal stem cells, and bone marrow-derived cells have been suggested as possible sources of myofibroblasts [8], [28], particularly in lung and kidney fibrosis [29], [30]. It has been demonstrated previously that myofibroblasts are responsible for contractures of fibrotic tissue, as characterized by strong ability of collagen synthesis, weak tensile strength and abilities of contraction, migration and extracellular matrix synthesis, as compared with fibroblasts [8]. Therefore, the myofibroblast is considered to be the most important cell in producing excessive extracellular matrix in fibrosis [31], [32]. In the current study, we observed that in vitro fibroblasts, treated by TGF-β1, had differentiated into myofibroblasts which appeared larger, spindle-shaped aligned in parallel sheets with dramatically increased α-SMA cytoskeleton expression. Similarly, we observed that α-SMA-positive myofibroblast cells increased significantly in silicotic nodules and fibrotic area in the silicotic lung. The increase in SMA-positive myofibroblasts in vivo and in vitro correlated with an enhanced expression of collagen types I and III, a finding in keeping with its preeminent role in development of lung fibrosis. Importantly, Ac-SDKP treatment markedly attenuated SMA-positive myofibroblast differentiation, as well as collagen deposition/expression in myofibroblasts induced by TGF-β1 and in the in vivo rat silicotic model.

SRF has been shown to regulate many genes involved in cellular proliferation, migration, differentiation, angiogenesis, and apoptosis. It is known to be upstream of α-SMA, and binding to the SRE (plasma response element) present in the α-SMA promoter activates the promoter of α-SMA mRNA [33]. In vitro studies have confirmed that the nuclear translocation of SRF can be induced by TGF-β, and that TGF-β up-regulates the expression of α-SMA in fibroblast to promote the differentiation from fibroblast to myofibroblast [9]. In the current study, we also observed that induction with TGF-β1 results in the up-regulation of TGF-β1 receptor (type I and II) and SRF. Pretreatment by LY364947, a specific TGF-β1 receptor inhibitor, was able to block the myofibroblast differentiation and down-regulate expression of TGF-β1 receptor and SRF. In vivo, the expression of SRF increased significantly and specifically in silicotic nodules and interstitial fibrotic tissue, similar to α-SMA. Ac-SDKP also down-regulated TGF-β and its receptors protein levels in the silicotic lung. Thus, by down-regulating both the ligand and receptor for TGF-β, Ac-SDKP is able to attenuate TGF-β signaling at multiple levels to prevent myofibroblast differentiation in rat silicotic model and in cultured fibroblasts. Activation of TGF-β receptors is known to lead to phosphorylation of receptor-activated Smad2/3, which forms a complex with Smad4. The activated Smad2/3-Smad4 complex translocates into the nucleus and activates target genes [34], [35]. Smad 7, an inhibitory Smad, associates stably with the TGF-β receptor complex and can inhibit TGF-β-dependent phosphorylation of Smad2/3. In previous work, we found that Ac-SDKP increases Smad7 levels and blocks Smad2/3 expression *in vivo*, which suggests that the anti-TGF-β effect of Ac-SDKP may involve Smad7-mediated inhibition [26].

It is well known that ACE, in addition to metabolism of AngI to AngII via its C-terminal domain, can also hydrolyze Ac-SDKP by its N-terminal catalytic domain [36], leading to Ac-SDKP inactivation. Furthermore, activation and production of AngII is associated with lung fibrosis which can be attenuated by AT_1_ blocker or ACE inhibitor [37], [38], [39]. In the present study, we observed that AngII level in plasma and lung tissue was up-regulated by silicosis, and similarly that ACE and AT_1_ in the lung were also elevated. Ac-SDKP did not significantly affect ACE or AngII levels, but did attenuate the silicotic-induced elevation of AT_1_. The inhibitory effect of Ac-SDKP effect on AT_1_ could contribute to Ac-SDKP’s anti-silicotic effect as the RAS system is well known to play a role in lung fibrosis.

Finally, an important caveat of our study is that Ac-SDKP was effective in attenuating myofibroblast differentiaton, collagen deposition, as well as TGF-β, SRF and AT_1_ expression in the silicotic lung when administered as a post-treatment or as pre-treatment. The pre-treatment approach involved administration of Ac-SDKP for two months in advance of induction of silicosis, whereas the post-treatment approach administered Ac-SDKP one month after silicosis induction. While both treatment approaches were effective, the prevention treatment approach was the most effective. Nevertheless, the fact that post-treatment with Ac-SDKP was effective is intriguing, and has importance for potential therapeutic application.

While this study has yielded important insights on the mechanisms underlying Ac-SDKP anti-fibrotic effects in the silicotic lung, some limitations exist. For instance, since ACE can hydrolyze and inactivate Ac-SDKP, better anti-fibrotic efficacy may be gained through use of Ac-SDKP analogues resistant to ACE metabolism. In addition, more studies are needed on the mechanisms of action of Ac-SDKP, including assessing effects upon the epithelium and/or macrophages so as to better understand its anti-fibrotic effect in silicosis. Furthermore, studies assessing Ac-SDKP effect on lung function are also needed. Finally, a significant unresolved issue is that no receptor for Ac-SDKP has been identified to date, and thus details concerning specific receptor mediation and associated signaling mechanisms continue to remain elusive. Thus, studies are urgently needed to address this vital issue.

### Conclusion

In conclusion, the results of the present study suggest a novel mechanism of action for Ac-SDKP’s beneficial effect in silicosis, which involves attenuation of TGF-β1 and its receptors, SRF and AT_1_ expression, collagen deposition, and myofibroblast differentiation. Since myofibroblast differentiation plays a critical role in the development of fibrosis in the silicotic lung, this novel regulatory effect of Ac-SDKP on myofibroblast differentiation is suggested to contribute significantly to its anti-fibrotic effect and its ability to decrease collagen deposition in the silicotic lung. The results further suggest that therapies targeting myofibroblast differentiation may have therapeutic efficacy in treatment of silicosis of the lung.
